# Wireless Neuromodulation for Chronic Back Pain: Delivery of High-Frequency Dorsal Root Ganglion Stimulation by a Minimally Invasive Technique

**DOI:** 10.1155/2017/4203271

**Published:** 2017-11-02

**Authors:** Bart Billet, Roel Wynendaele, Niek E. Vanquathem

**Affiliations:** ^1^AZ Delta Hospital, Roeselare, Belgium; ^2^StimRelieve LLC, Fort Lauderdale, FL, USA

## Abstract

**Objective:**

To evaluate the analgesic effect of a dorsal root ganglion (DRG) stimulation technology utilizing high-frequency pulse rates to treat intractable chronic back and leg pain.

**Methods:**

This case study presents the outcomes, with a novel, wireless, minimally invasive miniature neurostimulator system in a case of chronic back pain. The subject was implanted bilaterally with a Freedom 4A quadripolar electrode array at the L2 dorsal root ganglion. Stimulation was applied using 10 kHz pulse rate and 30 μs pulse width. A VAS pain-rating scale, Oswestry Disability Index (ODI), EQ-5D-5L Quality of Life Questionnaire 5 dimensions, and Patients' Global Impression of Change (PGIC) scale were evaluated at 12 weeks and 6 months post implantation.

**Results:**

VAS pain scores for back pain reduced from 91 to 31 mms and 80 to 35 mms for leg pain. Additionally, while stimulation remained paresthesia-free, there were a marked decrease in pain medications and an increase in quality of life. Also, an increase in functionality from crippled to moderate was reported. There were no adverse reactions related to the procedure or device.

**Conclusion:**

The minimally invasive, wireless approach to deliver high-frequency, paresthesia-free DRG stimulation for treatment of chronic back and leg pain associated with FBSS was effective and encouraging.

## 1. Introduction

Spinal cord stimulation (SCS) is a well-established therapy for FBSS and is widely accepted in terms of safety and efficacy [1–3]. Though studies have established the cost-effectiveness of SCS for FBSS, SCS has certain limitations, and only 50% of patients are able to report long-term success [4–6]. Reasons for inferior outcomes include device migration, ineffective stimulation parameters, device failure, and positional changes of the hardware. Positional changes can result in alterations of paresthesia distribution. The relative distance between the electrodes and spinal cord tracts can be different in certain positions (seated or lying down) due to the bulk of the battery or extensive wiring involved in an SCS operating system [2, 7]. These side effects may not be easily seen in the placement and/or displacement of the battery or electrodes.

Alternative techniques of stimulation and their targets are recommended to provide a better and more stable relief to intractable pain syndromes. The DRG is a cluster of cells inside the dura that transmits sensory information, including nociceptive signals, to the dorsal columns of the spinal cord. Studies have implicated the role of the DRG in the development and maintenance of chronic pain perception with demonstration of changes in cell membrane and gene expression [8, 9].

In cases not suitable for SCS, stimulation of the DRG may prove to be an important alternative. The bony encasement of the target and its stable anatomical position can mitigate issues, such as lead migration. The anatomical location of the DRG offers a closer proximity to the electrodes compared to the spinal cord and its dorsal columns. Thus, the stimulation parameters are expected to be more tolerable and lower in power consumption [10]. The wireless neurostimulation system presented in this report is aptly suited for overcoming the difficulties of the SCS while profiting from the benefits of DRG stimulation.

## 2. Materials and Methods

This is a case illustration of a patient with intractable chronic back pain following spine surgery.

### 2.1. Case Report

This subject had a traffic accident resulting in traumatic disc herniation at L5 and S1. A microdiscectomy was performed at L5 and S1. However, postoperative residual back pain remained, along with neuropathic pain on the right side along L5 dermatome distribution. As a result, an anterior lumbar interbody fusion was performed at L5 and S1, 6 months later. Nevertheless, the subject continued to have invalidating lumbar pain and persisting neuropathic pain along L5 dermatome on the right side. Interventional pain management was offered, as medical management (with tramadol HCl and paracetamol) failed to provide significant relief. Pulsed radiofrequency treatment followed by ablation of the facet joint did not provide relief. At this point, bilateral placement of DRG electrodes at L2 was offered.

### 2.2. Device Description

**Figure 1:**
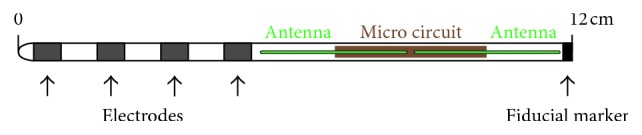
Neurostimulator electrode, MRI compatible, for both 1.5 and 3 Tesla (copyright StimRelieve LLC).

**Figure 2:**
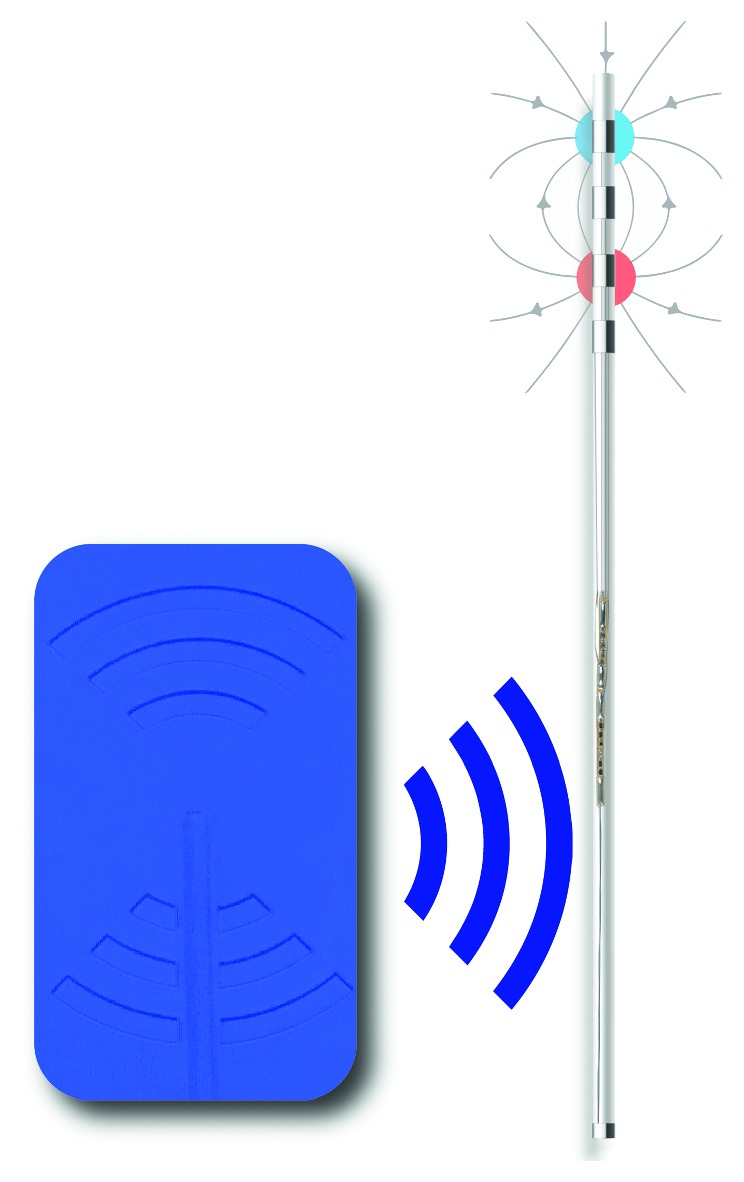
Neurostimulator receiver (copyright StimRelieve LLC).

**Figure 3:**
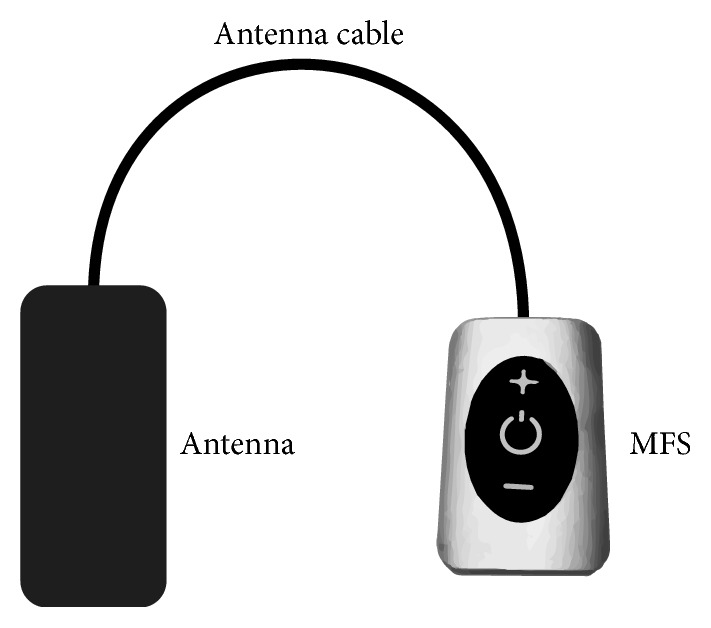
Freedom SCS external device (copyright StimRelieve LLC).

Refer to our earlier publication regarding the device [11]. The patient was implanted with two Freedom 4A electrode arrays, each array containing four contacts, at the L2 dorsal root ganglion under fluoroscopic guidance. The stimulator system utilizes an implantable passive electrode contact array, microprocessor receiver, and antenna embedded within the electrode wire that couples to an external transmitting antenna and external pulse generator (EPG) (Figures 1 and 2). The external transmitters (Figure 3) are worn by the patient over a single layer of clothing and are used to transmit power to the stimulator. The EPG is programmed by the clinician for the required stimulation parameters. The system uses 915 MHz radiofrequency energy for the transmission, and the distance between the implant and antenna remains short and the energy was relatively low. Wavelengths and product specifications have been designed to decrease risk related to the wireless transmission of energy [12] and reliably transfer the clinician's desired stimulation parameters with a wide available spectrum (amplitude: 1–24 mA; pulse width: 1–1000 microsec; frequency: 1–10,000 Hz).

### 2.3. Surgical Procedure

**Figure 4:**
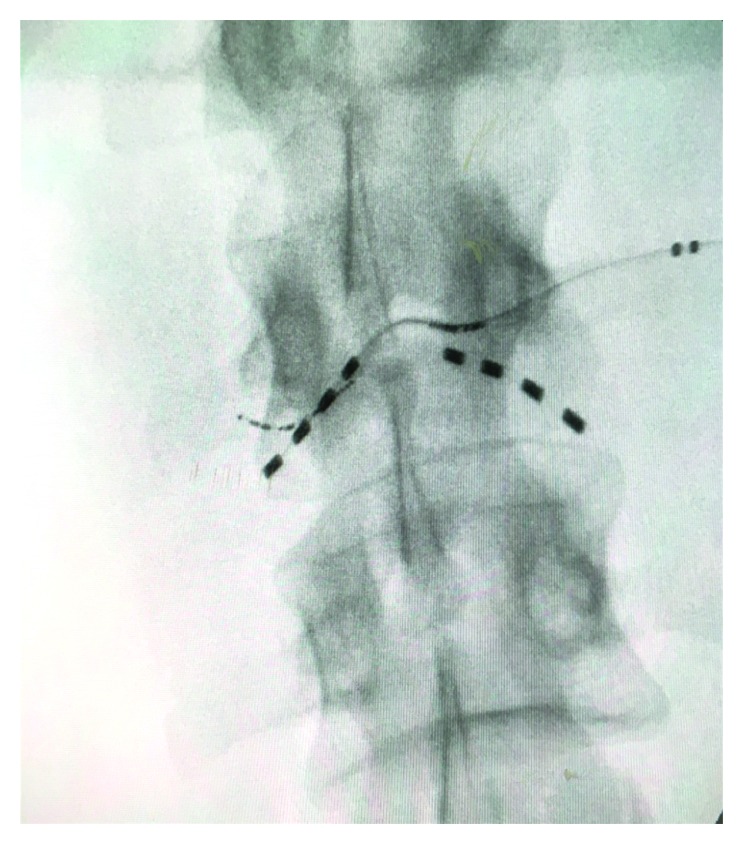
AP view of lumbar spine X-ray showing the electrode placement.

**Figure 5:**
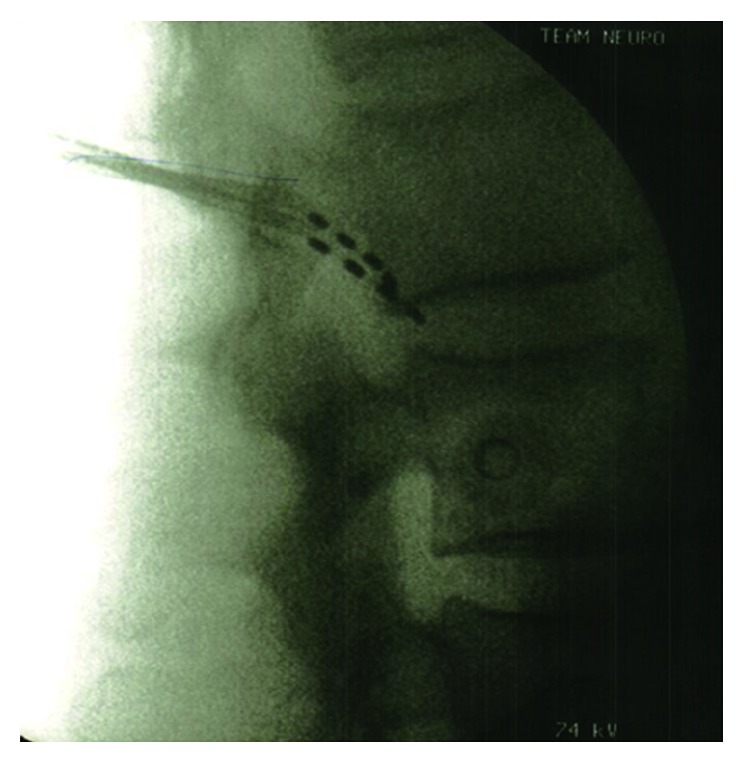
X-ray of lumbar spine, lateral view confirming the placement of electrodes.

Under strict aseptic precautions, the skin and subcutaneous tissues were infiltrated with local 1% lidocaine. A small skin incision was made for needle insertion, which was shaped by hand to match the contour to achieve appropriate electrode placement. Insertion of the lead was performed transverse and translaminar through 14 G Tuohy needle at L2 exiting nerve roots, coming from the cephalad end. Biplanar fluoroscopic images were used to monitor electrode positioning (Figures 4 and 5). The device was anchored via a subdermal suture located at the skin entry point. Distal tubing cut at the insertion point was buried subcutaneously, and the skin incision was closed.

### 2.4. Stimulation Protocol

Stimulation parameters were set at pulse widths of 30 microseconds and frequency of 10 kHz with intensity set between 1.5 and 2.5 mA for bilateral stimulation (both devices activated at the same time) with bipolar electrode selection, closest to the dorsal root ganglion. (This is not to be confused with the device communication frequency between the external generator and electrode microprocessor of 915 MHz.) During stimulation sessions, when therapy was needed to alleviate pain, patients wore an external transmitter over a single layer of clothing, positioned over the implant location of the electrode array (Figure 3).

## 3. Results

A VAS pain-rating scale, Oswestry Disability Index (ODI), EQ-5D-5L Quality of Life Questionnaire, and Patients' Global Impression scale (PGIC) were administered at 3, 5, 8, 12 weeks, and 6 months post implantation.

After the procedure, clinical response ensued in the form of 66% improvement for back pain and 56% for leg pain at 6-month follow-up as measured with VAS. There was a steady and progressive improvement of gait and posture. Functionality (ODI) improved with 61% from crippled to moderate disability. The patient reported a definite improvement on the PGIC (6 out 7) and an important increase in quality of life with the EQ-5D-5L (Table 1). Pain medication was reduced to tramadol HCl 37.5 mg twice daily.

## 4. Discussion

High-frequency stimulation at the DRG was effective in the treatment of pain associated with FBSS and for pain localized to the back and lower extremities. PROCESS reported that 50–60% of FBSS subjects achieved 50% or better leg pain relief through six months of SCS therapy [4, 13]. DRG stimulation seems to be better in terms of anatomical stability with lead migration of 3% (2 leads out of 67) [12], well below the 13.2% rate of migration for SCS with percutaneous leads placed over the dorsal columns, reported in a literature review of 51 studies [2], and 23% in a prospective study [14].

Strong supporters for DRG stimulation include its anatomical location that mitigates positional or postural influences on the device as well as the patient. There is also positive ability in the target region to induce stimulation coverage for those areas, which SCS would not be able to reach, for example, toes and foot [15, 16]. Additionally, the stimulation parameters require a lower demand in energy consumption.

In the present case illustration, we demonstrate the analgesic effect of high-frequency DRG stimulation, applying our minimally invasive placement of stimulation electrodes and wireless neuromodulation. This also supports and expands the number of indications for the treatment of other chronic pain conditions. There is no hardware and thus no complications related to its components. Percutaneous electrode placement devoid of any implanted pulse generator or the long connective wires can be advantageous to both patients and surgeons. They do not only add to comfort and cosmetics but also reduce costs, operating time, and postoperative pain, thus minimizing adverse events while achieving the desired pain control [17].

Larger prospective studies are required to further our knowledge about the wireless neuromodulation technology that has yielded promising results, thus far. We are in the process of evaluating the technology in other related areas of pain management also.

## Figures and Tables

**Table 1:** EQ-5D-5L.

Variable	Mobility	Self-care	Activity	Pain	Anxiety	State	EQ-VAS
Description	1 = no problems	1 = no problems	1 = no problems	1 = no pain	1 = not anxious	5-digit code for EQ-5D-5L	999 = missing value
	2 = slight problems	2 = slight problems	2 = slight problems	2 = slight pain	2 = slightly anxious		
	3 = moderate problems	3 = moderate problems	3 = moderate problems	3 = moderate pain	3 = moderately anxious		
	4 = severe problems	4 = severe problems	4 = severe problems	4 = severe pain	4 = severely anxious		
	5 = unable to	5 = unable to	5 = unable to	5 = extreme pain	5 = extremely anxious		
Baseline	4	4	4	4	3	44,443	20
6 months	2	2	2	3	2	22,232	73

## References

[1] Praeger J. (2010). Estimates of annual spinal cord stimulator implant rises in the United States. *Neuromodulation*.

[2] Cameron T. (2004). Safety and efficacy of spinal cord stimulation for the treatment of chronic pain: a 20-year literature review. *Journal of Neurosurgery*.

[3] Taylor R. S., Van Buyten J. P., Buchser E. (2005). Spinal cord stimulation for chronic back and leg pain and failed back surgery syndrome: a systematic review and analysis of prognostic factors. *Spine*.

[4] Kumar K., Taylor R. S., Jacques L. (2008). The effects of spinal cord stimulation in chronic pain are sustained: a 24-month follow-up of the prospective randomized controlled multicenter trial of the effectiveness of spinal cord stimulation. *Neurosurgery*.

[5] Kumar K., Hunter G., Demeria D. (2006). Spinal cord stimulation in treatment of chronic benign pain: challenges in treatment planning and present status, a 22-year experience. *Neurosurgery*.

[6] North R. B., Kidd D. H., Farrokhi F., Piantadosi S. A. (2005). Spinal cord stimulation versus repeated lumbosacral spine surgery for chronic pain: a randomized, controlled trial. *Neurosurgery*.

[7] Holsheimer J., Khan Y. N., Raza S. S., Khan E. (2007). Effects of electrode positioning on perception threshold and paresthesia coverage in spinal cord stimulation. *Neuromodulation*.

[8] Sapunar D., Kostic S., Banozic A., Puljak L. (2012). Dorsal root ganglion–a potential new therapeutic target for neuropathic pain. *Journal of Pain Research*.

[9] Van Zundert J., Patijn J., Kessels A., Lame I., van Suijlekom H., van Kleef M. (2007). Pulsed radiofrequency adjacent to the cervical dorsal root ganglion in chronic cervical radicular pain: a double blind sham controlled randomized clinical trial. *Pain*.

[10] Struijk J. J., Holsheimer J., van Veen B. K., Boom H. B. K. (1991). Epidural spinal cord stimulation: calculation of field potentials with special reference to dorsal column nerve fibers. *IEEE Transactions on Biomedical Engineering*.

[11] Perryman L. T., Speck B., Weiner R. L. (2017). A novel wireless minimally invasive neuromodulation device for the treatment of chronic intractable occipital neuralgia: case illustrations. *Journal of Neurology and Stroke*.

[12] Tyler Perryman L., Larson P., Glaser J. (2016). Tissue depth study for a fully implantable, remotely powered and programmable wireless neural stimulator. *International Journal of Nano Studies & Technology*.

[13] Liem L., Russo M., Huygen F. J. P. M. (2013). A multicenter, prospective trial to assess the safety and performance of the spinal modulation dorsal root ganglion neurostimulator system in the treatment of chronic pain. *Neuromodulation*.

[14] Kim D., Vakharyia R., Kroll H. R., Shuster A. (2011). Rates of lead migration and stimulation loss in spinal cord stimulation: a retrospective comparison of laminotomy versus percutaneous implantation. *Pain Physician*.

[15] de Andrade D. C., Bendib B., Hattou M., Keravel Y., Nguyen J.-P., Lefaucheur J.-P. (2010). Neurophysiological assessment of spinal cord stimulation in failed back surgery syndrome. *Pain*.

[16] Schu S., Gulve A., Eldabe S. (2014). Spinal cord stimulation (SCS) of the dorsal root ganglion (DRG) for groin pain—a retrospective review. *Pain Practice*.

[17] Yearwood T. L., Perryman L. T. (2015). Peripheral neurostimulation with a microsize wireless stimulator. *Progress in Neurological Surgery*.

